# Natural infections of highly pathogenic avian influenza virus H5N1 in wild birds between 2020 and 2023 in the UK: a retrospective study with focus on microscopic lesions, viral distribution and neurotropism

**DOI:** 10.1186/s13567-025-01656-z

**Published:** 2025-11-18

**Authors:** Bernat Martí-Garcia, Fabian Z. X. Lean, Alejandro Núñez, Natàlia Majó

**Affiliations:** 1 Pathobiology & Population Sciences, Royal Veterinary College, Hawkshead Lane, North Mymms, Hatfield, Hertfordshire, AL9 7TA UK; 2 Present Address: Department of Infectious Diseases and Public Health, Jockey Club College of Veterinary Medicine and Life Sciences, City University of Hong Kong, Kowloon, Hong Kong SAR China; 3 Department of Pathology and Animal Sciences, Animal and Plant Health Agency (APHA-Weybridge), Addlestone, UK KT15 3NB; 4 Unitat Mixta d’Investigació IRTA-UAB en Sanitat Animal, Centre de Recerca en Sanitat Animal (CReSA), Campus de la Universitat Autònoma de Barcelona (UAB), 08193 Bellaterra, Catalonia Spain; 5 Departament de Sanitat i Anatomia Animals, Facultat de Veterinària, Universitat Autònoma de Barcelona (UAB), Campus de La UAB, 08193 Bellaterra, Catalonia Spain

**Keywords:** Highly pathogenic avian influenza (HPAI), wild birds, neuropathology

## Abstract

Since the emergence of highly pathogenic avian influenza (HPAI) H5N1 clade 2.3.4.4 Goose/Guangdong lineage in Europe in 2014, an unprecedented outbreak occurred during the 2021–2022 epidemiological year, causing mass mortalities in wild birds, including birds of prey, but also increasingly affecting Charadriiformes, which has imposed substantial ecological and infection pressure at the wild-bird–poultry and avian–mammalian interface. Neurological signs have been associated with higher fatalities in birds but pathological examinations of natural cases, including viral distribution, are currently lacking. In this study, we have thoroughly assessed the histopathological lesions and antigen distribution by immunohistochemistry (IHC) from 115 PCR-positive wild birds that died naturally from HPAI, including Charadriiformes, birds of prey, gamebirds, waterfowl and captive wild birds. The commonest histological lesion was pancreatic necrosis followed by splenic necrosis, encephalitis or neuronal necrosis, myocardial necrosis or myocarditis, necrosis of the respiratory tract and hepatic necrosis. Overall, 96 birds tested positive by IHC in multiple organs and most of the viral antigen was detected in the brain followed by the respiratory tract, heart, pancreas and kidney. In the brain, viral antigen was most commonly detected in neurons, neuropil and endothelium. In conclusion, HPAI-associated mortality in different wild birds can be associated with multisystemic viral dissemination and tissue damage, with endothelial tropism being a key feature in neuroinvasion and disease pathogenesis.

**Supplementary Information:**

The online version contains supplementary material available at 10.1186/s13567-025-01656-z.

## Introduction

The emergence of the H5Nx clade 2.3.4.4 Goose/Guangdong (Gs/Gd) lineage in Europe has led to several epidemic waves involving domestic poultry and, more recently, mortalities in wild birds [1–3]. In 2020, the frequency of highly pathogenic avian influenza (HPAI) outbreaks caused by H5Nx increased dramatically, with broadening of susceptible avian species, which included Charadriiformes [2–10], and the inadvertent spillover to wild and/or domestic mammals with the heightened infection pressure [4]. The altered patterns of transmission accelerated genetic reassortment and led to emergence of new subtypes and genotypes, including the shift of H5N8 to the predominant and prevailing H5N1 [5–7], detection of a novel genotype EA-2023-DT (which seemed to transmit easier amongst gulls), EA-2024-DI and, more recently, EA-2021-I (H5N5). From late 2020, other wild birds, including birds of prey and seabirds, were also significantly affected, and other H5Nx viruses were incurred, including H5N3, H5N4 and H5N5 [6–9].

The prevailing circulating strains of H5N1 have been hypothesised to have arisen from a common ancestor that underwent multiple reassortment events, including strains from North Africa, the Middle East and southwest Asia [11, 12]. The global circulation of H5N1 has resulted in consequential disease, in particular in domestic poultry and wild birds between 2021 and 2023 [10, 13–18], with over 50 million birds culled in affected poultry establishments and over 13 000 detections in domestic and wild birds in Europe. Wild birds were particularly affected during the epidemic peaks (December 2021 to March 2023), which raised concerns for the conservation of certain species such as arctic skuas (*Stercorarius parasiticus*), Atlantic puffins (*Fratercula arctica*) and Dalmatian pelicans (*Pelecanus crispus*) [10, 13, 14]. These birds are of conservation concern as reported by the International Union for Conservation of Nature (IUCN).

During this period, the avian influenza (AI) wild bird passive surveillance scheme in Great Britain recorded a rise in HPAIV H5Nx-positive wild birds, particularly Charadriiformes, corvids, waterfowl and raptors [14]. These epizootic events in the wild bird population occurred simultaneously to numerous mortality outbreaks in the poultry sector, not only posing the wild bird-poultry interface at most risk but also favouring spillover to other mammals including humans [19]. In fact, during the 2021–2023 HPAI epidemics in Europe, the UK was one of the most severely affected regions, which can be explained by a combination of geographical, environmental and poultry demographic factors [20–23]. The UK is situated on a major migratory bird route, particularly for waterfowl and other species that travel between Europe and Africa, favouring viral spread over long distances [20]. In addition, in 2022, the UK experienced mild weather with warmer temperatures, which might have favoured viral persistence in the environment [21, 22]. The large wild bird mortalities observed in the UK would have facilitated viral incursion into poultry farms and high mortalities, with over 140 million birds in production in 2021 in the country [23]. Such a high density of poultry production would have favoured viral dissemination in intensively reared settings and put strict biosecurity measures at stake. In addition, considering the countless outbreaks also observed in poultry during this period, spill back from poultry to wild birds cannot be excluded [24].

Over the winter of 2021–2022, the first cases of HPAI in wild mammals were detected in foxes and seals in England and Sweden [7, 24]. Since then, multiple mammalian species have tested positive for HPAI including American minks, domestic cats, Eurasian otters and European badgers. Neurological and/or respiratory signs, along with significant mortalities, have been described in these cases [25–27]. In 2022, the incursion of H5N1 in an American mink farm in Spain represented the first and largest outbreak in Europe within captive conditions and was most likely mediated through gull birds given the high identity of the molecular profile of the virus [25]. Similarly, the 2023 outbreak in domestic cats in Poland was linked to avian strains that had been circulating in the country since the previous year. In both the mink and cat outbreaks, as well as in wild foxes and otters, the isolated viruses showed mutations in the PB2 protein, known as a molecular marker of adaptation to mammals [25–29]. Both in Europe and America, HPAI viruses have also been detected and associated with neurological disease and/or mortalities in marine mammals, including bottlenose dolphins [30], seals and sea lions [31, 32]. More recently, H5N1 has been detected in non-carnivorous mammals including goats, dairy cows from different farms across the USA [33, 34] and sheep in the UK. In ruminants, infections are mostly subclinical; however, cattle may show clinical signs such as general malaise, reduced milk production and mild respiratory signshttps://pmc.ncbi.nlm.nih.gov/articles/PMC12439802/ [35].

Interestingly, the bovine mammary gland has been detected as the main organ for viral shedding [36] and sporadic human cases have occurred, namely in people in close contact with these animals, raising concerns about the zoonotic potential of HPAI as an occupational hazard [33]. The continuous detection of HPAI viruses in wild and domestic mammals, either resulting from spillover from birds or spillback infection from other mammals, the rapid acquisition of viral mutations associated with mammalian adaptation, as well as the sporadic human infections with HPAI H5Nx viruses of clade 2.3.4.4b reported for the last two years, are all factors that highlight the need to intensify surveillance in mammals and humans [37].

Although pathological investigations of naturally occurring cases of HPAI in wild birds have been reported [38, 39], detailed descriptions of microscopic lesions and viral antigenic distribution using immunohistochemistry (IHC) in a large cohort of birds from broader taxa including shorebirds, birds of prey, waterfowl and free-range game birds, as well as detailed neuropathological investigations, are currently lacking despite neurological signs commonly reported in per-acute deaths. Therefore, the present work aims to (1) characterise the microscopic lesions of natural infections with H5N1 across a diverse range of wild birds (primarily from the UK), (2) characterise the viral distribution in tissues by IHC and (3) assess the viral distribution in the central nervous system (CNS), with the aim of providing preliminary insights into the neuroinvasion mechanisms in the affected wild bird population.

## Materials and methods

### Selection criteria

In total, 112 wild birds across Great Britain, submitted to the Animal and Plant Health Agency (APHA; UK) between 2020 and 2023 under the national avian influenza surveillance plan, were included. All included animals were PCR positive for H5N1 avian influenza virus (AIV) in the cloacal or oropharyngeal swab following described techniques [40–42]. Carcasses were received fresh or frozen–thawed for full post-mortem examinations. Macroscopic lesions were assessed and tissues collected for histopathological examination and immunohistochemical viral detection. The bird species comprised 33 Charadriiformes (17 skuas [8 *Stercorarius skua* and 9 *Stercorarius longicaudus*], 5 herring gulls [*Larus argentatus*], 4 black headed gulls [*Chroicocephalus ridibundus*], 3 gannets [*Morus bassanus*], a black backed gull [*Larus marinus*], a puffin [*Fratercula arctica*], a curlew [*Numenius arquata*] and a common gull [*Larus canus*]), 33 free-range Galliformes (27 pheasants [*Phasianus colchicus*] and 6 red-legged partridges [*Alectoris rufa*]), 23 birds of prey (8 common buzzards [*Buteo buteo*], 6 falcons [*Falco columbarius*], 2 Harris’s hawks [*Parabuteo unicinctus*], 2 sparrow hawks [*Accipiter nisus*], 2 goshawks [*Accipiter gentilis*], 2 white-tailed sea eagles [*Haliaeetus albicilla*] and a tawny owl [*Strix aluco*]), 11 waterfowl (4 Mallard ducks [*Anas platyrhynchos*], 3 mute swans [*Cygnus olor*], 3 Eurasian coots [*Fulica atra*] and a black swan [*Cygnus atratus*]), 7 Passeriformes (7 pied wagtails [*Motacilla alba*]) and 5 captive wild birds (4 Humboldt penguins [*Pheniscus humboldti*] and a pelican [*Pelecanus onocrotalus*]).

Data from 18/34 of the included Charadriiformes (8 great skua [*Stercorarius skua*], 1 long-tailed skua, 5 herring gulls and 4 black-headed gulls) has previously been published [38].

In addition, three ‘found dead’ wild birds submitted for routine diagnostic purposes to Servei de Diagnòstic en Patologia Veterinària (SDPV), Universitat Autònoma de Barcelona (UAB) in 2023 were also included in this study. These included a Charadriiforme (yellow-legged gull [*Larus michaellis*]) and 2 waterfowl (a Western cattle egret [*Bubulcus ibis*] and a grey heron [*Ardea cinerea*]).

### Histopathology and immunohistochemistry

Formalin-fixed tissues were processed for routine histologyinto paraffin blocks. Included tissues, where available, were heart (*n* = 107); skeletal muscle (*n* = 115); skin (*n* = 91), gastrointestinal tract (*n* = 105), including one or more of the following organs: proventriculus, gizzard, and small and large intestines; pancreas (*n* = 81); spleen (*n* = 76); kidney (*n* = 98); liver (*n* = 99); respiratory tract (*n* = 114), including one or more of the following organs: trachea and syrinx, lung, air sacs and nasal turbinate; brain (*n* = 110); reproductive organs (ovary or testes; *n* = 34); and eyes (*n* = 9).

Tissues were sectioned at 4 μm thickness and stained with haematoxylin and eosin for histological evaluation and immunohistochemical labelling using a mouse monoclonal immunoglobulin G1 (IgG1) antibody against the nucleoprotein of influenza A virus (Statens Serum Institute, Denmark; HYB 340–05) at 1:4000 for the detection of influenza viral antigen, as previously described [43]. Tissues were assessed on conventional light microscopy and histological and immunohistochemistry semi-quantitative scoring systems were applied as described by Landmann et al. 2021 [44]. Briefly, parenchymal necrotic/necrotizing inflammatory lesions in multiple influenza-target organs (heart, pancreas, spleen, brain, liver, kidney and lung) were scored as follows: 0 (absent), 1 (mild), 2 (moderate) and 3 (severe). A similar scoring for the distribution of avian AIV-antigen in parenchymal cells and parenchymal necrotic areas was applied as follows: 0 (absent), 1 (focal to oligofocal = less than 2% immunoreactive area for the spleen; less than a 5% immunoreactive area for the brain, lung, liver and pancreas; less than 10% immunoreactive area and less than two immunoreactive cells per high-power field for the heart; and less than two clusters per low power field or up to 2% of immunoreactive area for the kidney), 2 (multifocal = 5–20% immunoreactive area and up to five clusters per low-power field for the brain; 2–10 immunoreactive cells per high-power field for the heart; at least 3 immunoreactive cells per high-power field for the lung; 5–14% of immunoreactive area or 3–10 immunoreactive cells per high-power field for the liver; up to 5% immunoreactive area or at least two clusters per low-power field for the kidney; 5–24% of immunoreactive area or more than one cluster per low-power field for the pancreas; and 2–14% immunoreactive area or 16–40 immunoreactive cells per high-power field for the spleen) and 3 (coalescing to diffuse = more than 20% immunoreactive area and/or more than five clusters per low-power field for the brain; at least 10% immunoreactive area and/or more than 10 immunoreactive cells per high-power field for the heart; at least 5% immunoreactive area for the lung; at least 15% immunoreactive area and/or more than 10 immunoreactive cells per high-power field for the liver; 5% immunoreactive area with coalescing clusters or more than 5% immunoreactive area for the kidney; at least 25% immunoreactive area for the pancreas; and at least 15% immunoreactive area and/or more than 40 immunoreactive cells per high-power field for the spleen). In addition, topographical viral antigen distribution in the CNS was recorded, following the micro-anatomic nomenclature of the avian brain used by Chaves et al. [45, 46]: telencephalon (cerebral hemispheres), thalamus–midbrain and hindbrain (including brainstem and cerebellum).

### Statistical analysis

Descriptive statistics were computed on Microsoft Excel to quantify frequencies of histopathological lesions and immunohistochemistry labelling amongst the different tissues. Fisher’s exact tests and Chi squared tests, including graphs and illustrations, were performed using GraphPad Prism (version 10.2.3) to explore the relationships between (1) bird groups and most common histological lesions, (2) presence or absence of encephalitis and other histological lesions (i.e. pancreatic necrosis, splenic necrosis and myocardial necrosis), (3) type of bird and viral antigenic distribution, (4) type of bird and topographical viral distribution in the brain and (5) type of bird and viral cellular tropism in the CNS. Two-tailed Spearman correlation analyses were performed between histological and immunohistochemistry scores. Statistically significant *p*-values were expressed as *p < 0.05, **p < 0.01 and ***p < 0.001. Statistical analyses for Passeriformes, waterfowl, penguins and pelicans were omitted owing to the low number of cases.

## Results

### Histopathology

**Figure 1:**
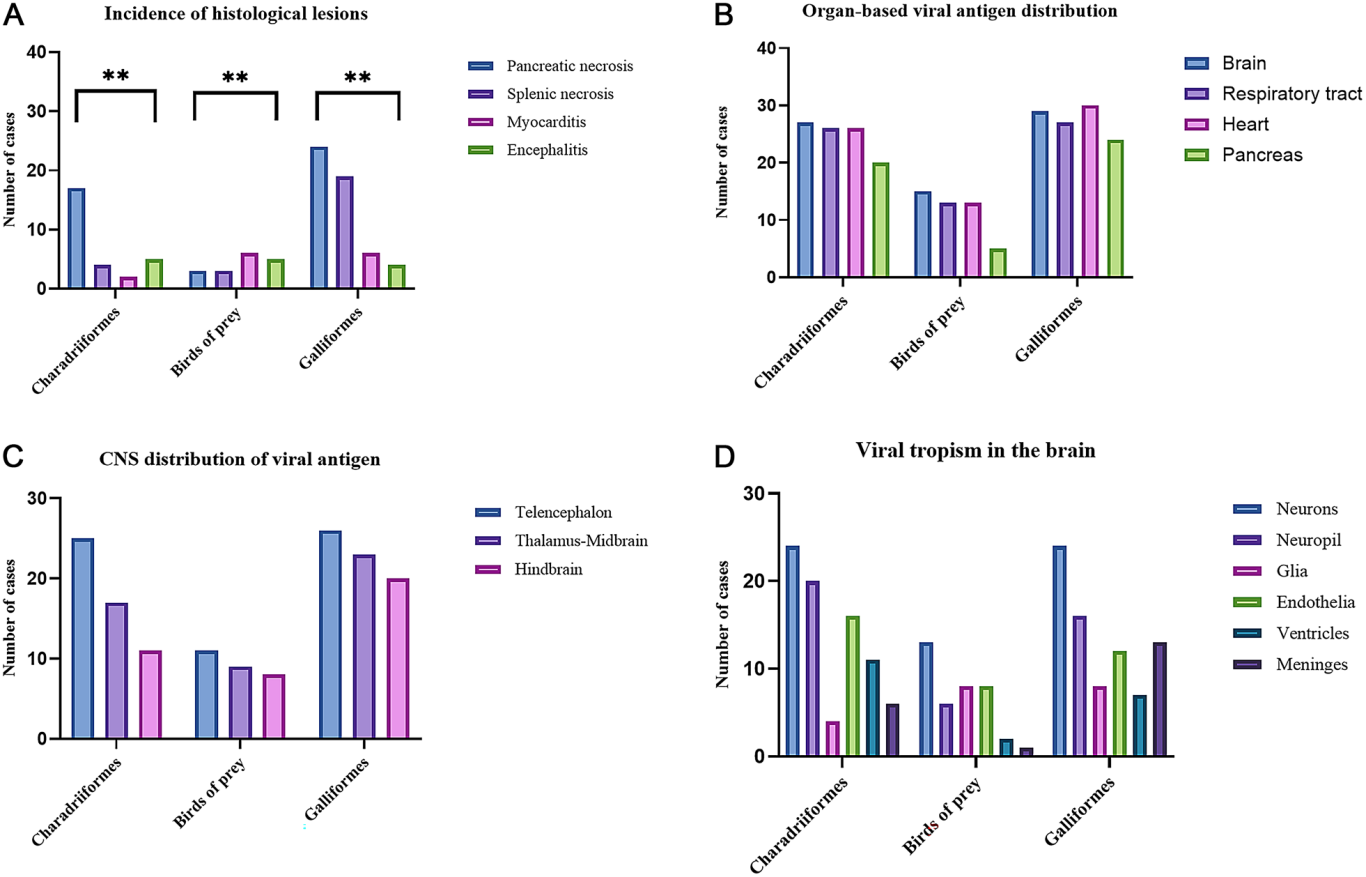
**Microscopic findings of wild birds infected naturally by H5N1.** Frequency of lesions in Charadriiformes, Galliformes and birds of prey (**A**). Pancreatic necrosis is the most common lesion in Charadriiformes and Galliformes, whilst myocarditis is slightly over-represented by the birds of prey. Splenic necrosis is mostly observed in Galliformes, and encephalitis predominates in birds of prey and Charadriiformes. Fisher’s exact test; **p < 0.01. Virus antigen distribution in tissue of Charadriiformes, Galliformes and birds of prey naturally infected with H5N1 (**B**). Most of the viral antigen is detected in the brain, followed by the respiratory tract, heart and pancreas in all bird groups. No statistically significant association is found between bird groups (*p* = 0.832; Chi-squared test). Topographic viral distribution in the CNS (**C**). The commonest area showing viral neurotropism is the telencephalon (cerebral hemispheres) in all bird groups, followed by the thalamus–midbrain and the hindbrain. No significant statistical relationship is observed between affected brain regions and included bird groups (*p* = 0.811; Fisher’s exact test). Cellular tropism in the CNS (**D**). Viral antigen is mostly observed in neurons and neuropil irrespective of the bird type, followed by endothelia, meninges, glia and ventricles. No significant statistical relationship is observed between cellular viral tropism in the brain and bird groups (*p* = 0.0896; Chi squared test).

**Figure 2:**
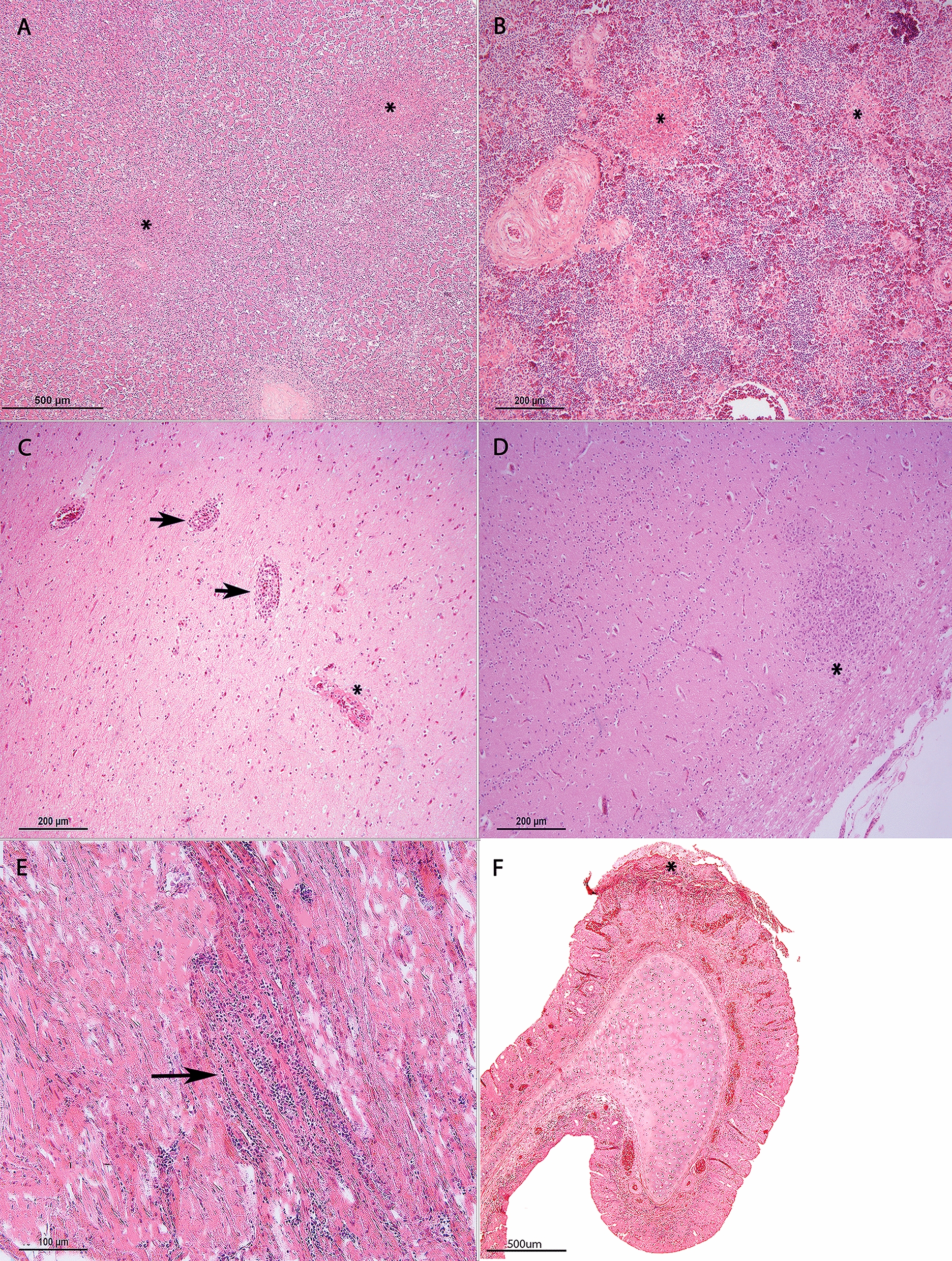
**Common histological lesions in the major organs of wild birds infected by H5N1. **Bifocal pancreatic necrosis (*) in a herring gull (*Larus argentatus*; **A**). Bifocal splenic necrosis (*) in a mute swan (*Cygnus olor*; **B**). Vasculitis (arrows) and fibrinoid necrosis (*) in the brain of a Harris hawk (*Parabuteo unicinctus*; **C**). Encephalitis with a glial nodule (*) in a herring gull (*Larus argentatus*; **D**). Lymphohistiocytic myocarditis (arrow) in a common buzzard (*Buteo buteo*; **E**). Focally extensive fibrino-necrotising rhinitis (*) in a red-legged partridge (*Alectoris rufa*; **F**).

The most common histological lesion in this study, irrespective of the avian taxa, was pancreatic necrosis (43.5%), followed by splenic necrosis (25.2%), encephalitis or neuronal necrosis (13.9%), myocardial necrosis or myocarditis (12.2%), necrosis of the respiratory tract (either in the nasal respiratory mucosa and/or trachea when available) (8.7%) or pulmonary parenchyma and hepatic necrosis (7.8%). Fisher’s exact test demonstrated a statistically significant relationship between the bird groups included in the analysis (Charadriiformes, birds of prey and Galliformes) and the four most frequently observed histological lesions including pancreatic necrosis, splenic necrosis, encephalitis and myocardial necrosis (*p* = 0.0037) (Figures 1A and 2). A summary of the data, including the frequencies of most common histological lesions, is provided in Additional file 1.

Pancreatic necrosis was observed in 24 Galliformes, 17 Charadriiformes, 3 birds of prey and 6 waterfowl, and was not observed in any of the Passeriformes, pelican or penguins. In most of the birds with pancreatic necrosis, the lesion was severe (76%), followed by moderate (18%) and mild in one mute swan (*Cygnus olor*).

Splenic necrosis was observed in 19 Galliformes, 3 birds of prey, 4 Charadriiformes and 3 waterfowl; spleen was not available in any of the Passeriformes. This lesion was severe in most of the cases (82.7%), followed by moderate severity (13.8%). Only a pheasant (*Phasianus colchicus*) showed mild splenic necrosis.

Encephalitis or neuronal necrosis was more commonly observed in birds of prey (21.7%) and waterfowl (15.4%), shortly followed by Charadriiformes (15.2%) and Galliformes (18.212.1%). These lesions were not observed in any of the pied wagtails, penguins or the pelican. The extent of encephalitic lesions was mostly moderate (56.3%), followed by severe (31.3%) and mild in 18.8% of the cases. When encephalitis and/or neuronal necrosis was observed alongside necrotic or inflammatory lesions in multiple organs, the heart (37.7%), pancreas (20%) and spleen (13.8%) were more commonly involved. However, this association was not statistically significant based on Fisher’s exact test (*p* = 0.0606) (Additional file 2).

Myocardial necrosis or myocarditis was more commonly observed in birds of prey (26.1%), followed by Galliformes (18.2%) and Charadriiformes (6.1%), and these lesions were moderate in 71.4% of cases, followed by mild (21.4%) intensity. No severe myocarditis was observed in this study.

Epithelial necrosis of the respiratory tract and hepatic necrosis were uncommon across all taxa. Necrosis of the respiratory tract epithelium was predominantly observed in waterfowl (15.4%), closely followed by Galliformes (15.2%) and birds of prey (4.3%). In the pelican (*Pelecanus onocrotalus*), a focal fibrino-necrotising tracheitis was observed. The necrosis of the respiratory tract in affected birds was mostly mild (60%), followed by moderate intensity (20%). Severe necrosis was observed only in the yellow-legged gull (*Larus michahellis*) (10%).

Three Charadriiformes, two Galliformes, two waterfowl, two penguins and one bird of prey (a falcon; *Falco columbarius*) showed hepatic necrosis; being moderate in 70% of cases, mild in 20% and of severe intensity in a single partridge (*Alectoris rufa*).

In only two birds, a herring gull (*Larus argentatus*) and a falcon (*Falco columbarius*), a focal area of necrosis with fibrin deposition and degenerated heterophils was observed in the feather follicle pulp cavity (pterylitis) (Figures 4A and B).

When all tissues were available for assessment, only nine birds did not show any histological lesions, including three Charadriiformes, three birds of prey, two Galliformes and a waterfowl. In contrast, when pancreas and/or spleen were not available, and no other lesions were observed, the number of birds demonstrating an absence of lesions raised to 27 individuals (7 Charadriiformes, 7 birds of prey, 7 Passeriformes, 4 waterfowl and 2 Galliformes). As incidental histological findings, one herring gull (*Larus argentatus*) and a skua (*Stercorarius skua*) showed endocardial fibroelastosis and endomyocardial fibrosis, respectively.

In 37.4% of the birds, at least two histological lesions were simultaneously detected, with the most common combination being pancreatic and splenic necrosis (45.2%).

### Viral antigen distribution across taxa

Overall, 96 birds (83.5%) were positive for Influenza A anti-nucleoprotein by IHC in one or multiple organs. IHC for viral antigen was negative in the following species: the western cattle egret (*Bubulcus ibis*), one common buzzard (*Buteo buteo*), one goshawk (*Accipiter gentilis*), the two white-tailed sea eagles (*Haliaeetus albicilla*), seven pied wagtails (*Motacilla alba*), the puffin *(Fratercula arctica*), the black backed gull (*Chroicocephalus ridibundus*), one mute swan (*Cygnus olor*) and the common gull (*Larus canus*). No histological lesions were observed in these birds. IHC was not available in three red-legged partridges (*Alectoris rufa*). A summary of the data, including the frequencies of organs most commonly positive by IHC, is provided in Additional file 1.

**Figure 3:**
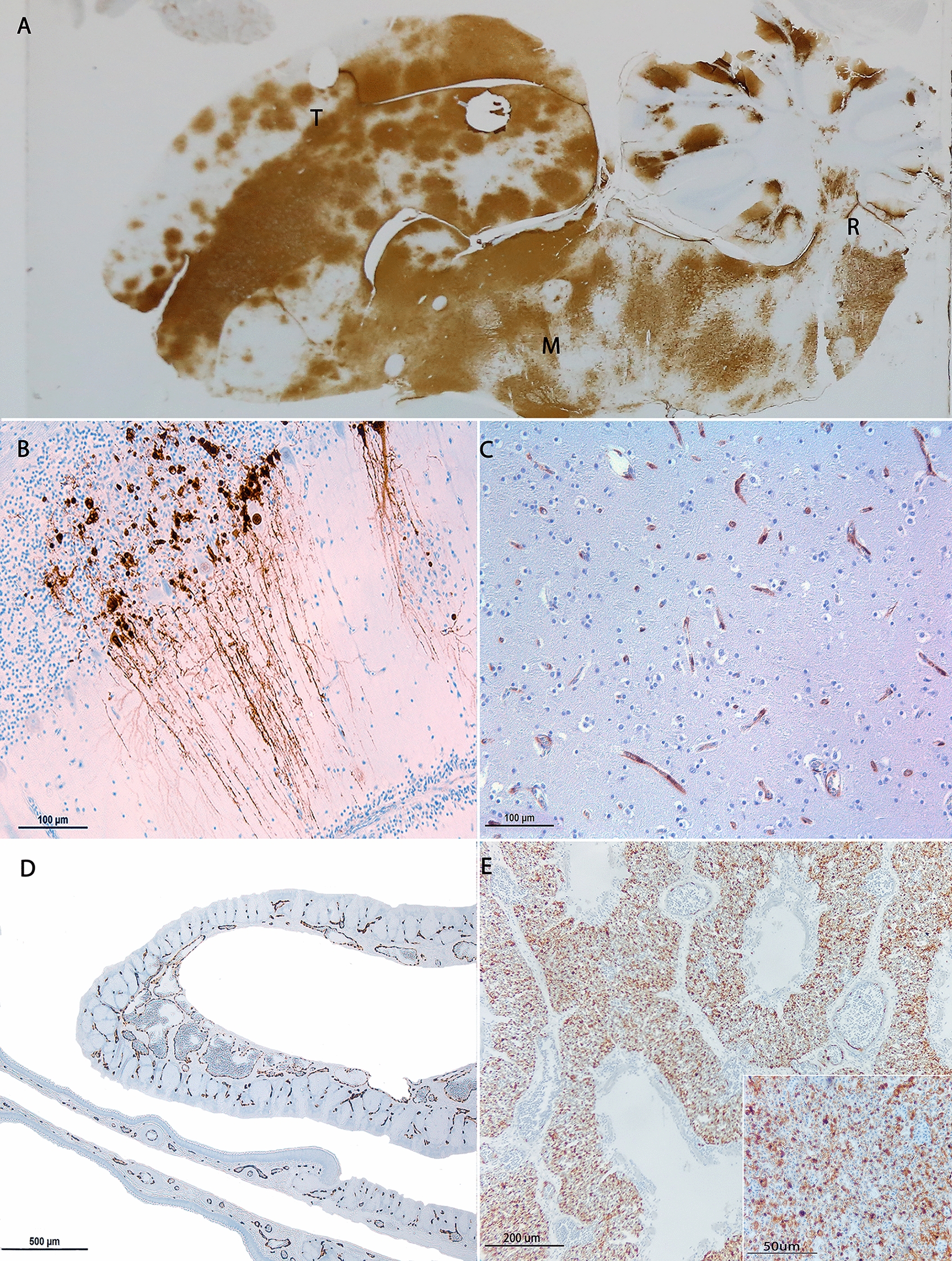
**Distribution and cellular tropism in the commonest organs showing H5N1 antigen by IHC.** Multifocal and generalised antigenic distribution in the brain of a pheasant (*Phasianus colchicus*) involving the telencephalon (T), midbrain (M) and rhombencephalon(R) (**A**). Intense cytoplasmic labelling in the Purkinje cells of a herring gull (*Larus argentatus*; **B**), brain endothelia of a grey heron (*Ardea cinerea*; **C**), nasal respiratory endothelia of a pheasant (*Phasianus colchicus*; **D**) and capillary bed of parabronchi in a Humboldt penguin (*Spheniscus humboldti*; **E**). The inset shows specific labelling of endothelial cells and interstitial round cells (likely of a monocyte–macrophagic lineage).

**Figure 4:**
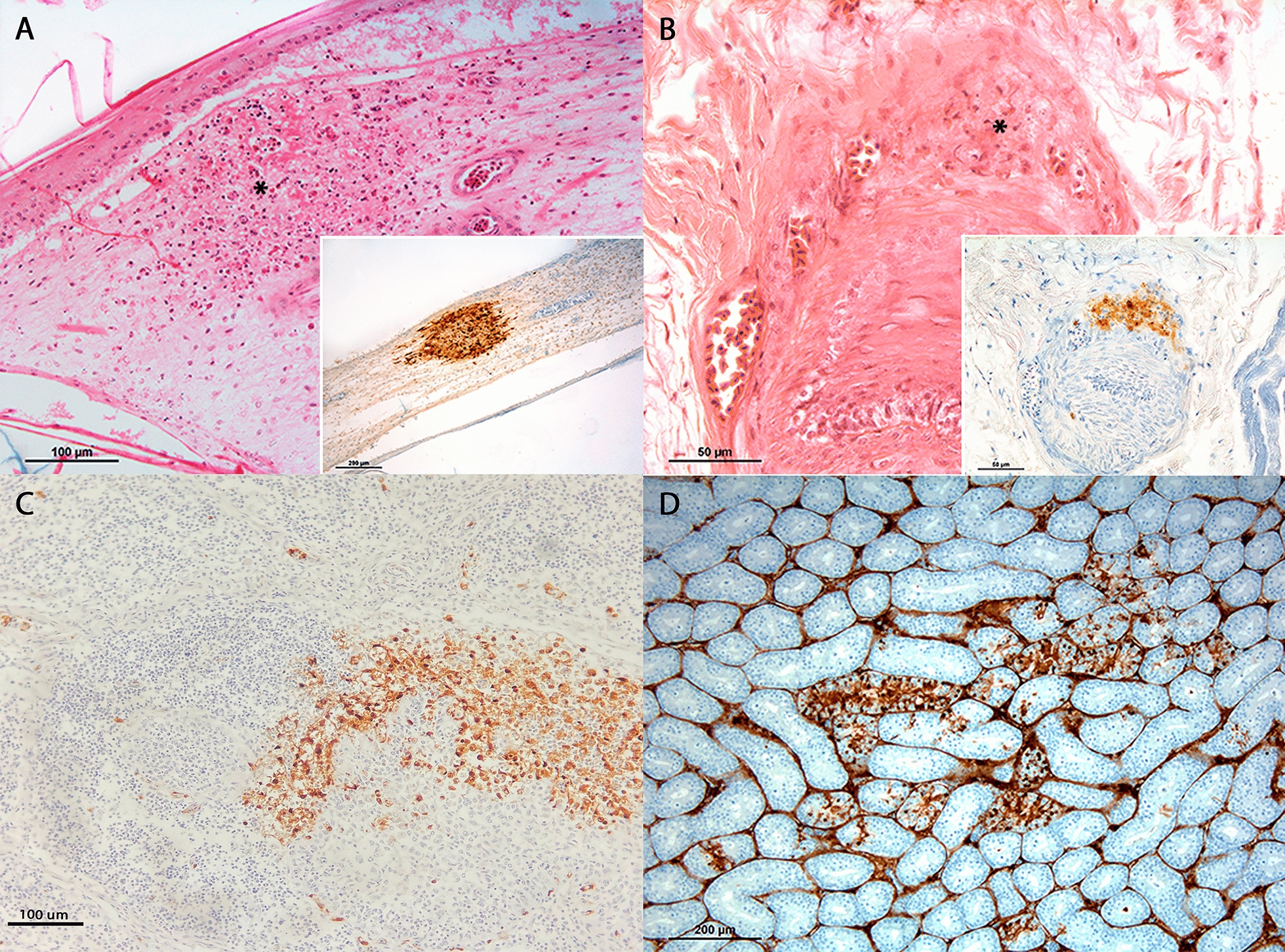
**Ancillary lesions and tropism of HPAI H5N1 in wild birds.** Fibrino-suppurative and necrotising pterylitis in a herring gull (*; **A**) and a falcon (*; **B**), with co-localisation of virus antigens by IHC (insets). Virus antigen detection in the testicles of a Humboldt penguin (**C**) and of a pheasant (**D**). In both cases, germ cells and interstitial capillaries demonstrate strong immunolabelling.

The distribution of the viral antigen per organ in the different bird taxa included in the statistical analyses is depicted in Figure 1B. Most frequently, viral antigen was detected in the brain (85.4%; Figures 3A–C) followed by the respiratory tract (80.2%; Figures 3D, E), heart (76%), pancreas (58.3%) and kidney (54.2%). Less commonly, viral antigen was observed in the liver (47.9%), gastrointestinal tract (39.6%), either epithelial or lymphoid-associated tissue, and spleen (39.6%). A large proportion of the birds showed immunopositivity within the respiratory tract (80.2%), with antigenic labelling in more than one anatomical location: the lungs (Figure 3E), including parabronchi, and trachea more frequently (66.2% for each location), followed by the respiratory nasal cavity (53.2%; Figure 4D) and air sacs (epithelia or endothelia; 51.9%). In 24.7% of the cases, only one respiratory structure displayed viral antigen by IHC, most commonly the lungs (52.6%), followed by the trachea (31.6%) and, to a much lesser extent, the respiratory nasal cavity (10.5%). Although less frequently observed, labelling was also detected in the reproductive tracts (25%; Figures 4C, D) and, when available, in the skin (11.5%; insets in Figure 4A, B).

Galliformes showed the highest frequencies of viral antigen in the heart (100%), brain (96.7%), respiratory tract (90%), pancreas (80%), kidney (66.7%), liver (63.3%) and gastrointestinal tract (60%). Charadriiformes showed the second highest frequency of antigenic labelling in multiple organs: brain (81.8%), heart and respiratory tract (78.8% each), kidney (63.6%) and pancreas (60.6%). Regarding birds of prey, viral antigen was more commonly observed in the brain (65.2%) and the respiratory tract and heart (53.5% each). Similarly, waterfowl showed most of the antigenic labelling in the brain and respiratory tract (61.5% each), followed by the pancreas (46.2%) and heart and liver (both 23.1%).

To assess the relationship between viral antigen abundance and the severity of microscopic lesions, Spearman correlation analyses were performed for Charadriiformes, birds of prey and Galliformes (Additional file 3). Across all three groups, the most consistent finding was a significant positive correlation between the abundance of viral antigen in the pancreas and spleen and the degree of necrosis and/or inflammation in these organs (*p* < 0.001). In birds of prey, additional significant positive correlations were noted between viral antigen abundance in the heart and liver and the degree of myocardial and hepatic necrosis, respectively (*p* < 0.01; Additional file 3). In contrast, no statistically significant correlations were identified for hepatic or cardiac pathology in Charadriiformes or Galliformes, or the encephalitis and respiratory lesions in association with the abundance of virus antigens.

Ten birds (10.4%) showed the viral antigen labelling only in one of the following organs: the heart (two gannets [*Morus bassanus*], a common buzzard [*Buteo buteo*] and a goshawk [*Accipiter gentilis*]), the brain (in the grey heron [*Ardea cinerea*], a long tailed skua [*Stercorarius longicaudus*] and a Humboldt penguin [*Pheniscus humboldti*]), the respiratory tract (in the Eurasian coot [*Fulica atra*] and the pelican [*Pelecanus onocrotalus*]) and kidney (in the sparrow hawk [*Accipiter nisus*]). Three pheasants showed viral antigen in all the assessed organs.

In the three organs that were more commonly positive by IHC, namely the brain, respiratory tract and heart, the viral antigen was frequently distributed in a coalescing-to-diffuse fashion (Figure 3) and, to a much lesser extent, followed a multifocal and, more rarely, oligofocal or focal distributions. In the rest of the assessed organs, the distribution patterns were similar.

Finally, uncommon immunopositive organs included reproductive organs (Figures 3 C, D), the skin (Figures 3 A, B insets) and eyes. In total, 22 birds (22.9%), including 11 Galliformes, 8 Charadriiformes, 2 birds of prey and a Humboldt Penguin, showed positive immunolabelling in the reproductive tracts (19 ovaries and three testicles). The skin showed positive immunolabelling in 11 birds (11.5%), including 5 Galliformes, 4 Charadriiformes, a falcon and a swan. Although eye samples were limited for assessment, they showed positive immunolabelling in the retina of two birds of this study: a Humboldt Penguin and a pheasant.

### Viral antigen distribution in the brain

Given the frequent ante-mortem neurological signs reported in wild birds infected with HPAI [11, 38] and the high detection rate of viral antigen in the CNS (Figures 1B and 3A–C), the topographical and cellular distribution of virus antigen in the brain was further characterised.

Galliformes and Charadriiformes showed the most frequent antigen labelling, followed by birds of prey and waterfowl. Topographically, telencephalon (cerebral hemispheres) was the most commonly affected region of the brain across avian taxa: Galliformes (31.7%), Charadriiformes (30.5%), birds of prey (13.4%) and waterfowl (9.7%). The thalamus–midbrain, with or without the optic tract, was immunolabelled in 26.8% of the Galliformes, 20.7% of Charadriiformes, 11% of birds of prey and 3.7% of waterfowl. In three Humboldt penguins, viral antigen was observed in the telencephalon, thalamus–midbrain and cerebellum. The hindbrain (including brainstem and cerebellum) was the least immunolabelled anatomical location in the CNS in all bird groups: Galliformes (24.4%), Charadriiformes (13.4%), birds of prey (9.7%) and only in one waterfowl: the grey heron (*Ardea cinerea*) (Figure 1C). While there was a higher incidence of viral antigen detected in the forebrain compared with the hindbrain, Fisher’s exact test did not reveal any significant statistical relationship between topographical viral distribution in the brain and the included bird groups in the analysis (*p* = 0.811).

The viral neurotropism was most commonly observed in neurons and neuropil irrespective of the bird group (82.3% and 59.7%, respectively) followed by endothelia (53.7%) (Figures 1D and 3B ,C). Meninges, glia and ventricles (ependyma/choroid plexus) displayed immunopositivity in the same proportions (26.8% each). In contrast to other species, the viral antigen in the brain of the three Humboldt penguins was exclusively detected in the endothelia. Galliformes, Charadriiformes, birds of prey and waterfowl displayed a similar cellular tropism, with neurons, neuropil and endothelia being the most frequently immunolabelled structures. The meninges were most frequently immunopositive in Galliformes (44.8%), the ependyma and/or choroid plexus in Charadriiformes (59.3%) and the glia in birds of prey (53.3%).

## Discussion

The present study provides an extensive characterisation of the microscopic lesions and viral antigen distribution in multiple organs of wild birds naturally infected with HPAI H5N1 clade 2.3.4.4b (Gs/Gd lineage) in the UK, and a subset of wild birds from Spain. The three wild birds from Spain included a yellow-legged gull, a Western cattle egret and a grey heron, which were not represented species in the UK AI wild bird passive surveillance scheme. To the best of the authors’ knowledge, this is the largest cohort of wild birds studied for this purpose in the UK. In addition, this work highlights the topological and cellular tropism of viral distribution in the brain to better understand the neuroinvasive mechanisms and neurotropism of this virus.

Since the initial HPAI outbreaks in the late 1990s and early 2000s, an expansion of susceptible species amongst birds has been observed, which may have been facilitated through adaptations of the Gs/Gd lineage in novel avian species beyond domestic Galliformes. This includes waterfowl and a variety of wild birds, such as Falconiformes, Accipitriformes and Charadriiformes, alongside increased neurological signs and lesions in the nervous system [10, 47]. Many of the reported natural and experimental infections with HPAI in birds identify the CNS as one of the primary target organs [48–50]. Recent studies have indicated a potential enhancement of adaptation of HPAI H5Nx viruses to wild birds, with these viruses being maintained in wild populations independently of domestic bird populations [49]. In addition, the H5 clade 2.3.4.4b viruses have shown novel epidemiological and pathobiological characteristics including variable pathogenicity in different duck species and mass mortalities in seabirds, as well as neurological signs and spillover to certain mammals [51]. Despite limited like-for-like comparisons between wild bird species and HPAI strains, both natural and experimental studies show similar spectra of lesions and viral distribution across a range of wild birds infected with different H5Nx clades. For example, pancreatic necrosis with abundant virus antigen is a common finding in natural infections with clades 2.2 and 2.3.4.4b in waterfowl and Charadriiformes, respectively [38, 52], as well as in experimental infections with clade 2.2 in common gulls (*Larus canus*) [53] and with clade 2.3.2.1 in birds of prey [54]. Encephalitis and myocarditis, with prominent antigen detection, were a common finding in experimentally infected herons with clade 2.3.2.1 [55]. Galliformes naturally infected with clade 2.3.2.1 showed necrosis of the respiratory tract epithelium, liver, pancreas and kidney [56], whereas experimental challenges in chickens with viruses of the clade 2.3.4.4 have shown similar histological lesions and viral antigen distribution in tissues, with the myocardium, lymphoid organs and the respiratory tract more commonly affected [57]. Interestingly, there were no macroscopic lesions in white-tailed eagles naturally infected with H5N1 2.3.4.4b, similar to that reported by Wünschmann et al. [39], but encephalitis was evident histologically. Viral antigen in these cases was detected in multiple organs, including spleen, brain and heart, and both neuro- and endotheliotropism were observed [58]. Overall, whilst there are inter-species lesional and viral antigenic variability in natural infection with HPAI H5N1 clade 2.3.4.4b, multi-organ tropism and, most importantly, neurotropism have been consistent features.

The precise neuropathogenesis of HPAI in birds remains poorly understood. In chickens experimentally infected with HPAI viruses, it has been shown that the virus reaches the CNS via the haematogenous route, disrupting the blood–brain barrier [45, 46]. Kim et al. [59] also observed viral antigen in the choroid plexus and ependyma, suggesting viral spread through the cerebrospinal fluid (), although whether this is the primary or secondary route of infection remains unclear. Interestingly, the works of Bertran et al. [60] and Chaves et al. [45] found that the olfactory route did not appear to be a portal of entry into the CNS. In addition, high viral antigen loads were detected by IHC and real time (RT)-PCR respectively, in orally infected falcons with H5N1, which developed fatal neurological disease [60]. This could suggest a haematogenous viral spread from the gastrointestinal tract to the CNS in this species.

In natural infections with HPAI in various Galliformes, raptors and waterfowl, neurological signs appeared shortly before mortalities occurred, suggesting a similar neuropathogenic mechanism [39, 61–63]. Brain histopathology in several bird types, including raptors, waterfowl and Galliformes revealed varying degrees of non-suppurative encephalitis, neuronal necrosis and gliosis, with abundant viral antigen detected in the brain using IHC and/or RT-PCR [39, 64, 65]. Despite the abundance of viral antigen in the CNS, no positive correlation was observed between antigen levels and the severity of encephalitis. This suggests that the extensive viral infection primarily induces functional or biochemical disturbances rather than overt morphological damage.

In the current study, the most common and statistically significant microscopic lesions observed across the most representative bird types (Galliformes, Charadriiformes and birds of prey) were pancreatic, splenic, neuronal necrosis and/or encephalitis and myocarditis, with these lesions being particularly severe in the pancreas and spleen. This pattern aligns with previous studies on natural H5Nx infections in wild birds [38, 39, 50, 63, 66]. In 35 cases, pancreatic and/or splenic necrosis were the only microscopic lesions, which should be considered during post-mortem sampling for diagnostic or surveillance purposes, as similar gross observations have also been noted [67]. Myocardial necrosis, the fourth most common lesion, was primarily moderate or multifocal and more frequently observed in Galliformes and birds of prey. These lesions have been reported in both experimentally infected Galliformes [68] and naturally infected birds of prey [39, 69].

Necrosis in the respiratory tract was infrequently observed and generally mild. Although this aligns with what is described elsewhere [39, 59], it could be possibly due to the rapid progression of the disease, which may have led to death before significant lesions could develop. Advanced autolysis and freeze–thaw artefacts could also have masked respiratory tract lesions. Notably, in this study, encephalitis and/or neuronal necrosis were almost always associated with multi-organ involvement, and although no statistically significant relationship was found between brain lesions and necrotic and/or inflammatory lesions in other organs (pancreas, spleen and heart), likely attributed to the sample size, a *p*-value close to significance was obtained in a Fisher’s exact test.

Viral antigen was detected in over 80% of the birds included in this study, even in cases where no histological lesions were observed, highlighting the high sensitivity of IHC to detect influenza antigens even in autolysed or poor-quality tissue samples [9, 38, 65, 70]. The brain, respiratory tract and heart were the most commonly positive organs, with a coalescing-to-diffuse distribution of viral antigen, indicating widespread infection. Although pancreatic necrosis was the most common histological lesion in this study, viral antigen was detected in around 60% of the cases. This discrepancy could be associated with a per-acute course and rapid clearance of the virus from the pancreas or, alternatively, viral antigens could have been masked/cleared by an advanced degree of autolysis of the samples and/or freeze–thaw artefacts. Galliformes and Charadriiformes showed the highest frequencies of positive labelling, likely reflecting their overrepresentation in this study. Importantly, Charadriiformes have been historically considered less susceptible to HPAI infection; however, findings from this study and others [38, 50] reflect a paradigm shift since 2020 with Gs/Gd clade 2.3.4.4.

The presence of viral antigen in the pancreas, heart and spleen was positively, strongly and significantly correlated with necrosis and/or inflammation in these organs as examined across the Charadriiformes, birds of prey and Galliformes, consistent with previous reports [38, 39, 50]. In addition, in birds of prey, viral antigen in the liver was positively, strongly and significantly correlated with hepatic necrosis, which together with viral splenotropism and splenic necrosis (features rarely described in raptors [39, 58]), emphasise the consideration of the spleen for diagnostics and surveillance post-mortem examinations and sampling. In Charadriiformes and Galliformes, viral antigen in the pancreas and spleen showed positive, strong and significant correlation with necrosis in these organs, as previously reported [38, 63].

In the brain, the viral antigen was most commonly detected in the cerebral hemispheres (telencephalon), followed by the thalamus–midbrain and hindbrain. When viral neurotropism was assessed at a cellular level, neurons and neuropil were the most frequently immunolabelled structures, followed by endothelia. Altogether, these findings suggest that the virus could have a primary neuronal tropism for sensory, more specialised neurons and possibly reaching the brain via systemic vascular spread. The involvement of meninges, glia and ependyma could further support a systemic viral endotheliotropism leading to CNS infection, potential disruption of the blood–brain barrier [46] and subsequent neuroparenchyma spread through trans-synaptic spread, leading to tissue damage and dysfunction.

In the respiratory tract, the lungs and trachea were the most frequently immunopositive organs, followed by the respiratory nasal cavity and air sacs. There is contrasting information about viral antigen distribution in the respiratory tract of natural infections with HPAI in different birds. None or rare viral antigen was found in the respiratory tract of wild raptors [39, 58], pheasants [67] or buzzards [49], whereas the Hsueh et al. study [71] found immunopositive signal in the lung in nearly 80% of the included wild birds. Regarding experimental infections, viral antigen has been found in the respiratory tract of chickens [45, 57], ducks [72] and, rarely, falcons [60]. In the current study, the low availability of nasal cavity, particularly fresh samples and those containing specialized olfactory epithelium, was a major limiting factor to more exhaustively assess viral distribution in the respiratory tract and neuroinvasion route through the olfactory nerve.

The reproductive tract of HPAI infected wild birds is inconsistently, if not rarely examined. In this study, viral antigen was detected in the ovaries and, to a lesser extent, in the interstitium and germinal cells of the testis of the nearly 23% positive reproductive tracts. While H5N1 viral antigen has been previously observed in the ovaries of Galliformes, Charadriiformes and birds of prey [38, 65, 71], this is the first report of antigen detection in the testis of wild Galliformes and a penguin. This finding suggests that the testis may be an overlooked tissue affected by systemic viral endotheliotropism. Viral entry may breach the blood–testis barrier to reach germinal cells, or a localised innate antiviral response may impair spermatogenesis and androgen production, as observed in other viral testicular infections [73]. However further studies are needed to clarify the mechanisms. The potential implications of HPAI infection of the gonads for these birds’ reproductive performance remains unclear considering the severe and acute clinical course of disease, which is invariably fatal.

In a small subset of cases (~10%), primarily Galliformes, viral antigen was also detected in the skin, particularly in the feather follicle epithelium. This has been previously described in pheasants [70], ducks and geese [74], and gulls [38], as part of H5Nx pantropism. Notably, the feather epithelium has been implicated in viral dissemination, and feathers have shown better sensitivity than other swab types for real time quantitative PCR (RT-qPCR) testing [74]. The current work also describes the presence of viral antigen in the skin of herring gulls, falcons, mute swans and a curlew for the first time.

There are several limitations of the study, these include that most of the animals were found dead in the wild, limiting the quality of histopathological interpretation and viral antigen detection. The unknown age and breeding status of the birds also adds variability in disease outcome and interpretation of the significance of lesions, particularly with reproductive organs. In addition, the number of included birds varied largely across the different groups, with Galliformes being over-represented compared with others. Further, as the study included cases of natural infection with H5N1 HPAI, it does not provide insight into the dynamics of the infection. Lastly, routine testing in wild bird surveillance schemes include only swabs from oropharyngeal and/or cloacal orifices for PCR testing, hence tissue RT-qPCR was unavailable for the current analysis. The authors acknowledge the likelihood of PCR-positivity in the antigen-negative cases; however, direct correlations between tissue viral RNA loads and viral antigen loads and distribution in tissues could not be assessed in our case.

In conclusion, this work highlights pancreatic, splenic and neuronal necrosis and/or encephalitis as the most common microscopic lesions owing to naturally acquired infections with HPAIV H5N1 clade 2.3.4.4b in the largest cohort of wild birds in the UK, including a great proportion of Charadriiformes, which since the 2020 epidemic wave, should be considered of interest for virus surveillance. Viral antigen was detected in the brain, respiratory tract, heart and pancreas more commonly and its detection was positively correlated with necrotic and/or inflammatory lesions in the pancreas, heart, spleen in all bird groups and, importantly, the liver in birds of prey. In the brain, neurons, neuropil and endothelia were the most frequently immunolabelled in all bird groups. The mortality is hence associated with multisystemic dissemination of virus and resultant tissue damage and endothelial tropism is a key feature in the pathogenesis of the natural infection. The most likely neuroinvasive mechanism is the vascular route, with rapid parenchymal tropism and spread, although further studies are warranted to fully elucidate the neuropathogenesis and potential for viral spread through various, perhaps overlooked tissues, such as reproductive organs and the skin.

## Supplementary Information

**Additional file 1.** **Data summary, including main histological lesions and most frequently labelled organs by (IHC) per bird family.**

**Additional file 2. Association between encephalitis and other lesions in wild birds naturally infected with H5N1.** Encephalitis and/or neuronal necrosis is observed alongside necrotic or inflammatory lesions in multiple organs, including the heart (37.7%), pancreas (20%), and spleen (13.8%) more commonly. However, this association is not statistically significant based on Fisher’s exact test (*p* value = 0.0606).

**Additional file 3. Histopathological and immunohistochemistry scoring correlations in wild birds naturally infected with H5N1 Across all species.** (**A**), and consistently within subsets of Charadriiformes (**B**), birds of prey (**C**), and Galliformes (**D**), the abundance of viral antigen in the pancreas and spleen is statistically significantly and positively correlated with the degree of necrosis and/or inflammation in the corresponding organs. In birds of prey (C), additional moderate, statistically significant positive correlations were observed between viral antigen abundance in the heart and liver, and the degree of myocardial and hepatic necrosis, respectively. Spearman two-tailed analysis. *p < 0.05, **p < 0.01, ***p < 0.001. Grey-shaded areas indicate analyses that could not be performed due to low sample size; white-shaded cells represent values approximating a Spearman correlation coefficient of 0.

## Data Availability

No datasets were generated or analysed during the current study.
