# Supplementary material for: Natural infections of highly pathogenic avian influenza virus H5N1 in wild birds between 2020 and 2023 in the UK: a retrospective study with focus on microscopic lesions, viral distribution and neurotropism
Source: Vet Res. 2025 Nov 18;56:218. doi: 10.1186/s13567-025-01656-z (PMC12625443; doi:10.1186/s13567-025-01656-z)
Supplement: Supplementary file 1 — Additional file 1. Data summary, including main histological lesions and most frequently labelled organs by (IHC) per bird family. [file 13567_2025_1656_MOESM1_ESM.docx]

**Additional file 1. Data summary (main histological lesions and most frequently labelled organs by immunohistochemistry (IHC)) per bird family.**

|  | **Lesions** | | | **Viral distribution by IHC** | | |
| --- | --- | --- | --- | --- | --- | --- |
| **Order/family** | Pancreatic necrosis | Splenic necrosis | Encephalitis | Brain | Respiratory tract | Heart |
| **Charadriiformes (n=34)** |  |  |  |  |  |  |
| *Stercorarius* spp. (n=17) | 11/17 | 2/17 | 1/17 | 15/17 | 16/17 | 15/17 |
| *Larus argentatus* (n=5) | 5/5 | 1/5 | 2/5 | 5/5 | 5/5 | 5/5 |
| *Chroicocephalus ridibundus* (n=4) | N/A | N/A | 0/4 | 4/4 | 4/4 | 3/4 |
| *Morus bassanus* (n=3) | N/A | N/A | 1/3 | 1/3 | 0/3 | 3/3 |
| *Larus marinus* (n=1) | N/A | N/A | N/A | N/A | 0/1 | N/A |
| *Larus canus* (n=1) | 0/1 | 0/1 | 0/1 | 0/1 | 0/1 | 0/1 |
| *Larus michaellis* (n=1) | 1/1 | 0/1 | 1/1 | 1/1 | 0/1 | 0/1 |
| *Fratercula arctica* (n=1) | N/A | N/A | 0/1 | 0/1 | 0/1 | 0/1 |
| *Numenius arquata* (n=1) | N/A | 1/1 | 0/1 | 1/1 | 1/1 | 0/1 |
| **Galliformes (n=33)** |  |  |  |  |  |  |
| *Phasianus colchicus* (n =27) | 19/27 | 17/27 | 3/27 | 26/27 | 24/27 | 27/27 |
| *Alectoris rufa* (n=6) | 5/6 | 2/6 | 1/6 | 3/6* | 3/6* | 3/6* |
| **Birds of prey (n=23)** |  |  |  |  |  |  |
| *Buteo buteo* (n=8) | N/A | 0/8 | 3/8 | 5/8 | 4/8 | 6/8 |
| *Falco columbarius* (n=6) | 3/6 | 1/6 | 1/6 | 6/6 | 5/6 | 3/6 |
| *Parabuteo unicinctus* (n=2) | 0/2 | 1/2 | 1/2 | 2/2 | 2/2 | 1/2 |
| *Accipiter nisus* (n=2) | N/A | N/A | 0/2* | 1/2* | 1/2 | 1/2 |
| *Accipiter gentilis* (n=2) | N/A | N/A | 1/2 | 0/2 | 0/2 | 1/2 |
| *Haliaeetus albicilla* (n=2) | N/A | 0/2 | 0/2 | 0/2 | 0/2 | 0/2 |
| *Strix aluco* (n=1) | N/A | N/A | 0/1 | 1/1 | 1/1 | 1/1 |
| **Waterfowl (n=13)** |  |  |  |  |  |  |
| *Anas platyrhynchos* (n=4) | 3/4 | 1/4 | 0/4 | 4/4 | 3/4 | 0/4 |
| *Cygnus olor* (n=3) | 2/3 | 1/3 | 0/3 | 1/3 | 2/3 | 2/3 |
| *Fulica atra* (n=3) | 0/3 | N/A | 0/3 | 1/3 | 2/3 | 1/3 |
| *Cygnus atratus* (n=1) | N/A | N/A | 1/1 | 1/1 | 1/1 | N/A |
| *Bubulcus ibis* (n=1) | 0/1 | 0/1 | 0/1 | 0/1 | 0/1 | 0/1 |
| *Ardea cinerea* (n=1) | 1/1 | 1/1 | 1/1 | 1/1 | N/A | 0/1 |
| **Other (n=12)** |  |  |  |  |  |  |
| *Motacilla alba* (n=7) | 0/7* | N/A | 0/7 | 0/7 | 0/7 | 0/7 |
| *Pheniscus humboldti* (n=4) | 0/4 | 0/4 | 0/4 | 3/4 | 2/4 | 1/4 |
| *Pelecanus onocrotalus* (n=1) | N/A | N/A | 0/1 | 0/1 | 1/1 | N/A |

N/A: tissue not available

*Tissue not available in half or more of the animals.
