# Supplementary figures and images for: Natural infections of highly pathogenic avian influenza virus H5N1 in wild birds between 2020 and 2023 in the UK: a retrospective study with focus on microscopic lesions, viral distribution and neurotropism
Source: Vet Res. 2025 Nov 18;56:218. doi: 10.1186/s13567-025-01656-z (PMC12625443; doi:10.1186/s13567-025-01656-z)

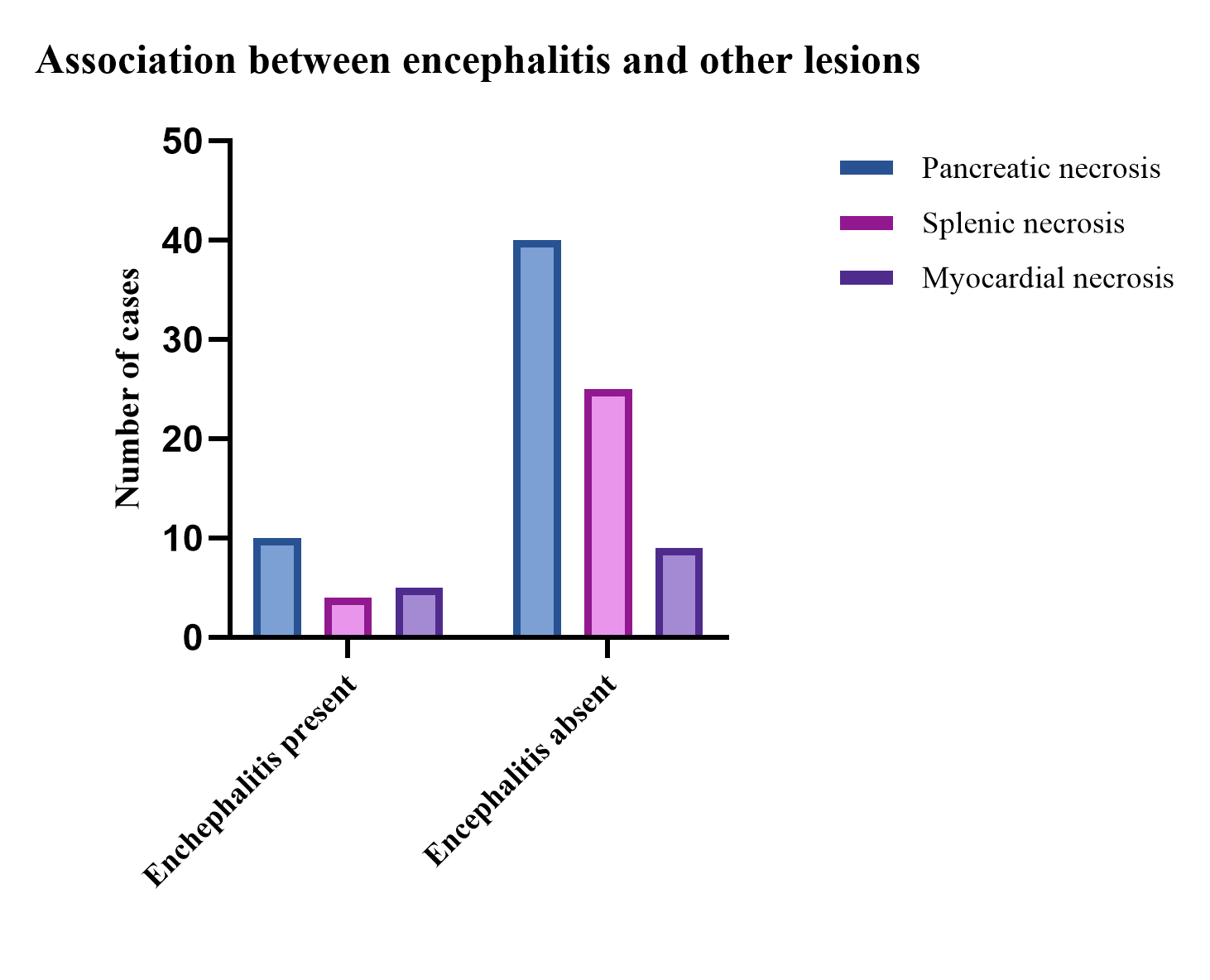

Supplement: Supplementary file 2 — Additional file 2. Association between encephalitis and other lesions in wild birds naturally infected with H5N1. Encephalitis and/or neuronal necrosis is observed alongside necrotic or inflammatory lesions in multiple organs, including the heart (37.7%), pancreas (20%), and spleen (13.8%) more commonly. However, this association is not statistically significant based on Fisher’s exact test (p value = 0.0606). [file 13567_2025_1656_MOESM2_ESM.tif]

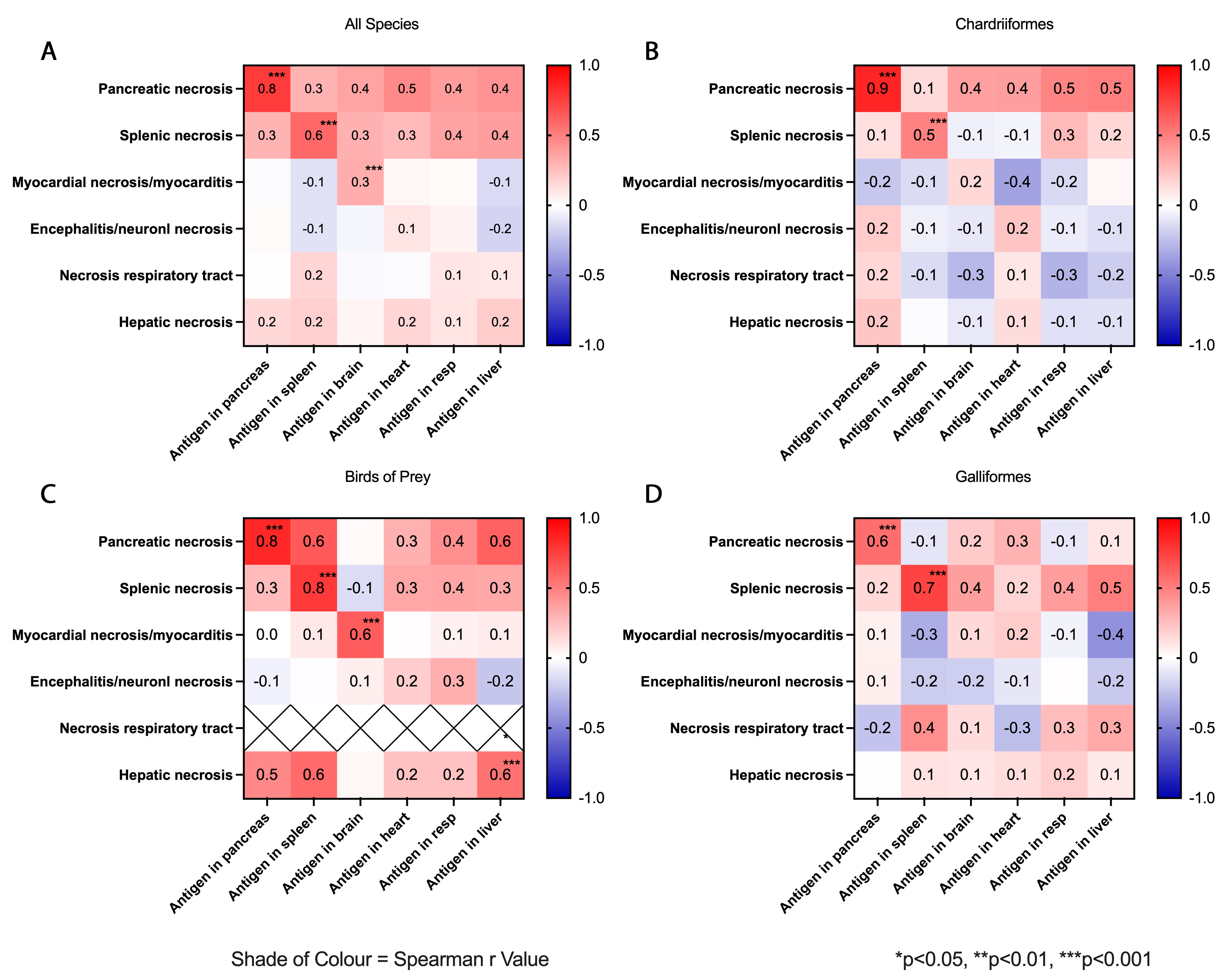

Supplement: Supplementary file 3 — Additional file 3. Histopathological and immunohistochemistry scoring correlations in wild birds naturally infected with H5N1 Across all species. (A), and consistently within subsets of Charadriiformes (B), birds of prey (C), and Galliformes (D), the abundance of viral antigen in the pancreas and spleen is statistically significantly and positively correlated with the degree of necrosis and/or inflammation in the corresponding organs. In birds of prey (C), additional moderate, statistically significant positive correlations were observed between viral antigen abundance in the heart and liver, and the degree of myocardial and hepatic necrosis, respectively. Spearman two-tailed analysis. *p < 0.05, **p < 0.01, ***p < 0.001. Grey-shaded areas indicate analyses that could not be performed due to low sample size; white-shaded cells represent values approximating a Spearman correlation coefficient of 0. [file 13567_2025_1656_MOESM3_ESM.tif]
